# A *Phaseolus vulgaris* Extract Reduces Cue-Induced Reinstatement of Chocolate Seeking in Rats

**DOI:** 10.3389/fphar.2016.00109

**Published:** 2016-04-26

**Authors:** Irene Lorrai, Valentina Piga, Mauro A. M. Carai, Antonella Riva, Paolo Morazzoni, Gian Luigi Gessa, Giancarlo Colombo, Paola Maccioni

**Affiliations:** ^1^Neuroscience Institute, National Research Council of Italy, Section of CagliariCagliari, Italy; ^2^Cagliari Pharmacological Research s.r.l.Cagliari, Italy; ^3^Indena S.p.AMilan, Italy

**Keywords:** *Phaseolus vulgaris* dry extract (Beanblock^®^), chocolate-flavored beverage, reinstatement of seeking behavior, relapse-like behavior, rats

## Abstract

Previous evidence has suggested that treatment with a standardized dry extract of *Phaseolus vulgaris* reduced intake and operant self-administration of highly palatable foods and fluids in rats and mice. The present study was designed to assess whether such extract was also effective in reducing seeking behavior for a highly hedonic chocolate-flavored beverage, using a “reinstatement” procedure adopted from the drug addiction research field and modeling relapse behavior. Rats were initially trained to lever-respond for the chocolate-flavored beverage under the Fixed Ratio (FR) 10 schedule of reinforcement. Subsequently, rats were exposed to an extinction responding phase, during which lever-responding – being unreinforced – diminished progressively up to extinction. Lever-responding was then powerfully reinstated by the non-contingent presentation of a complex of gustatory, olfactory, auditory, and visual stimuli previously associated to the availability of the chocolate-flavored beverage. Acute, intragastric administration of *P. vulgaris* dry extract (100 and 500 mg/kg) reduced lever-responding by 40–45%, in comparison to vehicle condition. These results indicate the ability of *P. vulgaris* dry extract to reduce seeking behavior for a highly palatable nourishment in an experimental model of relapse into disordered eating of palatable foods. The unavailability of the chocolate-flavored beverage in the reinstatement session tends to exclude that the observed effect of the *P. vulgaris* dry extract was secondary to any inhibition of carbohydrate metabolism; conversely, it is the likely consequence on a central action on the rewarding and hedonic properties of food.

## Introduction

The *Phaseolus vulgaris* genus includes all species of legume seeds typically referred to as common beans. Nowadays, *P. vulgaris* is considered a major functional food: extracts from *P. vulgaris* have indeed been repeatedly reported to reduce (i) food intake, body weight, lipid deposit, and glycemia in a series of validated animal models of overeating, obesity, diabetes, and metabolic syndrome (e.g., Grant et al., 1995; Pusztai et al., 1995, 1998, 2008; Bardocz et al., 1996; Tormo et al., 2004; Carai et al., 2009, 2011; Fantini et al., 2009; Loi et al., 2013) and (ii) appetite, food intake, body weight, waist circumference, and glycemia in healthy, overweight, or obese individuals (e.g., Layer et al., 1985; Boivin et al., 1987; Udani et al., 2004; Celleno et al., 2007; Udani and Singh, 2007; Rondanelli et al., 2011; Spadafranca et al., 2013). Together, these results suggest that *P. vulgaris* extracts may constitute a valuable source for novel and potentially effective therapies for obesity and related disorders (see Obiro et al., 2008; Astell et al., 2013; however, see also Onakpoya et al., 2011).

Recent studies from this lab have suggested that extracts from *P. vulgaris* may preferentially suppress several behaviors motivated by highly palatable nourishments in rodents: administration of standardized extracts of *P. vulgaris* indeed suppressed intake of butter cookies, condensed milk, and chocolate-flavored beverages in rats (Carai et al., 2009; Fantini et al., 2009) and mice (Loi et al., 2013) as well as operant self-administration of a chocolate-flavored beverage in rats (Maccioni et al., 2010). When the study design envisaged concurrent availability of the highly palatable food and regular food pellets, *P. vulgaris* extracts were found to be more potent and effective in suppressing intake of the palatable over regular food (Fantini et al., 2009), suggesting that *P. vulgaris* extracts may have a greater impact on the hedonic, rather than nutritive, properties of food.

With the aim of characterizing more in-depth the pharmacological profile of *P. vulgaris* extracts, the present study was designed to investigate the effect of a *P. vulgaris* extract on seeking behavior for a highly palatable food in rats. To this end, we employed (i) the same, standardized dry extract of *P. vulgaris* tested in all previous studies with different palatable nourishments (Carai et al., 2009; Fantini et al., 2009; Maccioni et al., 2010; Loi et al., 2013) and (ii) a chocolate-flavored beverage possessing high hedonic, reinforcing, and motivational properties in rats (see Maccioni and Colombo, 2016); additionally, we adapted to the chocolate-flavored beverage an experimental procedure, named “reinstatement of seeking behavior,” largely used in the drug addiction field to model relapse episodes and loss of control over substances of abuse (see Markou et al., 1993; Bossert et al., 2013) and, in much fewer instances, palatable foods (Martín-García et al., 2011; Calu et al., 2014). In this procedure, laboratory animals are initially trained to lever-respond for a given substance under standard schedules of reinforcement. Once lever-responding has stabilized, animals are exposed to an extinction responding phase, during which lever-responding – being unreinforced – diminishes progressively up to virtually complete extinction. After extinction, lever-responding (or seeking for the substance) is resumed, or reinstated, by different stimuli, including (i) limited, non-contingent availability of the substance, (ii) olfactory or visual cues previously associated to the substance (predictive of its availability), (iii) exposure to stressful stimuli, and/or (iv) injection of specific drugs. Concurrence of these stimuli or events is expected to trigger an intense reinstatement of lever-responding (still unreinforced), proposed to mimic human craving and seeking for the specific substance (see Markou et al., 1993; Bossert et al., 2013; Calu et al., 2014).

The procedure of reinstatement of seeking for a chocolate-flavored beverage, recently set up in this lab (see Maccioni and Colombo, 2016), is based on the ability of a complex of gustatory, olfactory, auditory, and visual stimuli to promptly and effectively resume previously extinguished lever-responding for the chocolate-flavored beverage in rats. With relevance to the aims of the present study, reinstatement of seeking behavior for the chocolate-flavored beverage was found to be pharmacologically manipulable: for instance, the cannabinoid CB_1_ receptor antagonist/inverse agonist, rimonabant, known to suppress appetite and food intake in laboratory animals and humans (see Carai et al., 2006; Leite et al., 2009; Scherma et al., 2014), suppressed the reinstatement of seeking behavior for the chocolate-flavored beverage in rats (Maccioni and Colombo, 2016).

## Materials and Methods

The experimental procedure employed in the present study was in accordance with the Italian Law on the “Protection of animals used for scientific reasons.”

### Animals

Adult, male Wistar rats (*n* = 30; Harlan Laboratories, San Pietro al Natisone, Italy), weighing approximately 300 g at the start of the study, were used. Rats were housed 3 per cage. All cages had wood chip bedding. The animal facility was under an inverted 12:12 h light-dark cycle (lights on at 8:00 p.m.), constant temperature of 22 ± 2°C, and relative humidity of approximately 60%. Standard rat chow and tap water were always available in the homecage, except as noted below. Rats were extensively habituated to handling and intragastric infusion (by means of a stainless steel gavage).

### Extract Preparation

Preparation procedure of *P. vulgaris* dry extract (Beanblock^®^) has been described in detail elsewhere (Fantini et al., 2009). Briefly, *P. vulgaris* dry extract was prepared by means of aqueous extraction and alcoholic precipitation from the common kidney bean. Bean extract was obtained by extraction with citrate buffer and precipitation with ethanol. The obtained extract was characterized by a standardized composition in: (i) 8.5% (w/w) α-amylase inhibitor, with inhibiting activity of 1,400 U/mg; (ii) phytohaemoagglutinin (haemoagglutinating activity equal to 16 HAU/mg).

### Chocolate-Flavored Beverage

The chocolate-flavored beverage was prepared diluting powdered Nesquik^®^ (Nestlè^®^ Italiana, Milan, Italy) in tap water. Concentration of Nesquik^®^ chocolate powder was 5% (w/v) throughout the study. This concentration was selected on the basis of the results of previous experiments in which it was largely preferred over a wide range of concentrations (see Maccioni and Colombo, 2016). The chocolate-flavored beverage was prepared daily and sipper bottles (see below) were shaken immediately before the start of each session to prevent any powder deposit. The chocolate-flavored beverage provided 0.8 kJ/g.

Previous experiments demonstrated that this chocolate-flavored beverage possesses highly hedonic, reinforcing, and motivational properties in rats (e.g., Maccioni et al., 2008, 2010; Zaru et al., 2013a,b).

### Apparatus

Operant sessions were conducted in modular chambers (Med Associates, St. Albans, VT, USA), located in sound-attenuated cubicles, with fans for ventilation and background white noise. The front panel of each chamber was equipped with (i) one retractable response lever, (ii) one green stimulus light mounted above the lever, (iii) one sonalert, and (iv) the retractable spout of a liquid sipper bottle (250-ml capacity) located outside the chamber. A white house light was centered at the top of the back wall of each chamber.

Start of the self-administration session was signaled by illumination of the house light and insertion of the lever. Achievement of the response requirement (RR; see below) resulted in (i) exposure of the sipper bottle spout inside the operant chamber, (ii) illumination of the green stimulus light, and (iii) activation of the sonalert; all these three events lasted 5 s. End of the self-administration session was associated to retraction of the lever and switch off of the house light.

### Experimental Procedure

A detailed description of the experimental procedure used in the present study has recently been given elsewhere (see Maccioni and Colombo, 2016).

#### Training and Maintenance Phases

To facilitate the acquisition of lever-responding behavior, rats were water-deprived in their homecage over the 12 h preceding the first two operant sessions. Self-administration sessions were conducted daily, 5 days per week (Monday to Friday), during the first half of the dark phase of the light/dark cycle. Self-administration sessions lasted 60 min. During the first two sessions, rats were trained to lever-respond on a Fixed Ratio (FR) 1 (FR1) schedule of reinforcement for the chocolate-flavored beverage. FR was progressively increased from FR1 to FR10 over 10 sessions. Subsequently, 20 additional self-administration sessions with FR10 were conducted (maintenance phase), so that number of lever-responses for and intake of the chocolate-flavored beverage stabilized in all rats before the start of the test sessions.

#### Extinction Responding Phase

Rats were divided into three groups of *n* = 10, matched for body weight and number of lever-responses for the chocolate-flavored beverage over the last five self-administration sessions of the maintenance phase. At the end of the maintenance phase, rats of all three groups underwent an extinction responding phase, during which daily sessions – lasting 60 min and occurring with no weekend interruptions – were characterized by unavailability of the chocolate-flavored beverage; specifically, sipper-bottle delivery system, green stimulus light, and sonalert were off, and lever-responding was unreinforced. An extinction criterion was set at ≤ 30 lever-responses per session for three consecutive sessions.

#### Reinstatement session

The day after achievement of the extinction criterion, each rat was exposed to a single reinstatement (test) session, during which a stimulus complex – previously associated to availability of the chocolate-flavored beverage – was presented. This stimulus complex was composed of: (i) click emitted by the introduction, into the operant chamber, of the sipper bottle spout; (ii) turning on of the green stimulus light; (iii) activation of the sonalert; (iv) availability of the chocolate-flavored beverage for 5 s. This stimulus complex was presented for 10 times within 100 s. Immediately after the 10th presentation of the stimulus complex, the lever was introduced inside the chamber, the house light was switched on, and lever-responses were recorded. Lever-responding during the reinstatement session was unreinforced.

*Phaseolus vulgaris* dry extract was suspended in distilled water with 0.5% methylcellulose and administered intragastrically (infusion volume: 4 ml/kg), at doses of 0, 100, and 500 mg/kg, 120 min before the start of the reinstatement session. Dose-range of *P. vulgaris* dry extract and pretreatment time were chosen on the basis of results of previous studies from this lab (e.g., Maccioni et al., 2010).

### Measured Variables and Data Analysis

In the maintenance phase, measured variables were (i) number of lever-responses for the chocolate-flavored beverage and (ii) amount of self-administered chocolate flavored beverage [expressed in ml/kg and determined by weighing the sipper bottle (0.01-g accuracy) before and after the session]. Data on number of lever-responses over the entire 20-session phase and the last 5-session period were analyzed by separate 1-way ANOVAs with repeated measures.

In the extinction responding phase, the measured variable was the number of sessions needed to achieve the extinction criterion. These data were analyzed by 1-way ANOVA and Log Rank test.

In the reinstatement session, the measured variable was the number of lever-responses. Data on the effect of *P. vulgaris* dry extract on this variable were analyzed by a 2-way [phase (extinction/reinstatement); treatment (dose of *P. vulgaris* dry extract)] ANOVA with repeated measures on the factor “phase,” followed by the Newman–Keuls test for *post hoc* comparisons.

## Results

### Maintenance Phase

**FIGURE 1:**
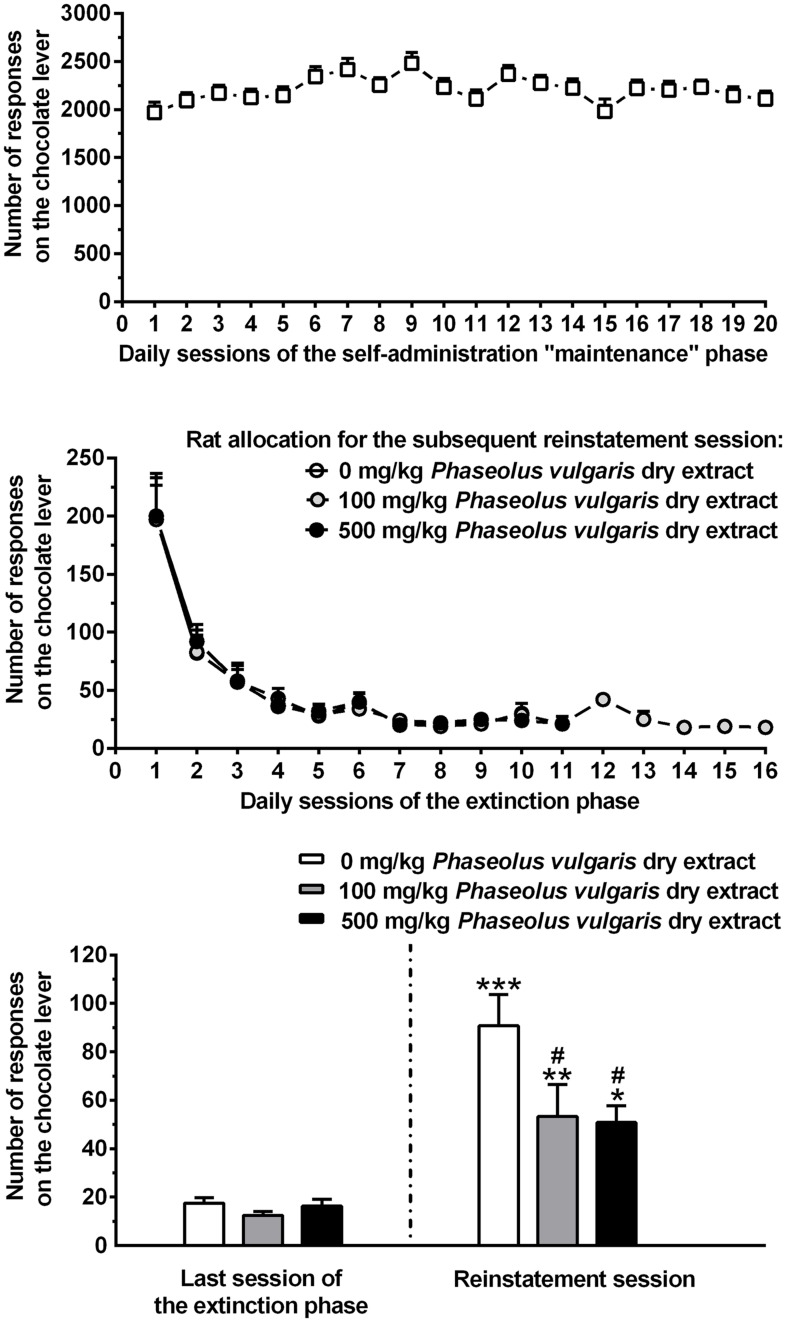
**(Top)** The self-administration behavior of a chocolate-flavored beverage in Wistar rats. Rats were trained to lever-respond for the chocolate-flavored beverage [5% (w/v) Nesquik^®^ chocolate powder in water] under the Fixed Ratio (FR) 10 schedule of reinforcement in daily 60 min self-administration sessions. During these 20 daily self-administration sessions (maintenance phase), self-administration behavior stabilized in all rats. Each point of this panel is the mean ± SEM of *n* = 30 rats. **(Center)** The extinction responding behavior. The extinction responding phase occurred after the self-administration maintenance phase. During the sessions of the extinction responding phase, lever-responding was unreinforced (the chocolate-flavored beverage was unavailable). Based on the number of lever-responses for the chocolate-flavored beverage over the last five sessions of the maintenance phase, rats were divided into the three groups (*n* = 10) that – after completion of the extinction responding phase – received the three different doses of *Phaseolus vulgaris*. Each point of this panel is the mean ± SEM of sample sizes varying between 1 and 10, depending on the session in which each single rat achieved the extinction criterion (i.e., ≤30 lever-responses per session for three consecutive sessions). **(Bottom)** The effect of treatment with different doses of a dry extract of *P. vulgaris* (Beanblock) on reinstatement of seeking for the chocolate-flavored beverage. This test session occurred once each single rat had achieved the extinction criterion. In the reinstatement session (lasting 60 min and occurring the day after the extinction responding phase), unreinforced lever-responding was reinstated by the repeated presentation of a complex of gustatory, olfactory, auditory, and visual stimuli previously associated to availability of the chocolate-flavored beverage. *P. vulgaris* dry extract was administered intragastrically, at doses of 0, 100, and 500 mg/kg, 120 min before the start of the session. Each bar of this panel is the mean ± SEM of *n* = 10 rats. ^∗^*P* < 0.05, ^∗∗^*P* < 0.01, and ^∗∗∗^*P* < 0.0005 in comparison to the same rat group in the last session of the extinction responding phase (Newman–Keuls test); ^#^*P* < 0.005 in comparison to the vehicle-treated rat group in the reinstatement session (Newman–Keuls test).

Over the 20 daily self-administration sessions of the maintenance phase (occurring in between training and extinction responding phases), lever-responding for the chocolate-flavored beverage under the FR10 schedule of reinforcement was extremely high, averaging approximately 2200 responses/session (**Figure 1**, top). These data confirm that this chocolate-flavored beverage had strong reinforcing properties in rats (Maccioni and Colombo, 2016). Over the entire 20-session phase, mean lever-responding for the chocolate-flavored beverage displayed some inter-session variability [*F*(19,551) = 5.28, *P* < 0.0001] (**Figure 1**, top), that vanished completely over the last five sessions [i.e., the time period used for rat allocation to the three different experimental groups (see above); *F*(4,116) = 1.75, *P* > 0.05] (**Figure 1**, top). Amount of self-administered chocolate-flavored beverage averaged approximately 75 and 70 ml/kg/session over the entire 20-session phase and the last 5-session period, respectively.

### Extinction Responding Phase

Log Rank test indicated that the profile of lever-responding over the extinction responding phase did not differ among the three rat groups subsequently treated with 0, 100, and 500 mg/kg *P. vulgaris* dry extract (χ^2^ = 2.064, *P* > 0.05; **Figure 1**, center). Additionally, the three rat groups did not differ in number of sessions of the extinction responding phase needed to achieve the extinction criterion [7.2 ± 0.6, 9.4 ± 1.2, and 8.1 ± 0.7 (mean ± SEM) in rats subsequently treated with 0, 100, and 500 mg/kg *P. vulgaris* dry extract, respectively, *F*(2,27) = 1.64, *P* > 0.05].

### Reinstatement Session

ANOVA indicated a significant effect of presentation of the stimulus complex previously associated to the chocolate-flavored beverage [*F*(1,54) = 54.81, *P* < 0.0001] and of treatment with *P. vulgaris* dry extract [*F*(2,54) = 4.35, *P* < 0.05], as well as a significant interaction between the two factors [*F*(2,54) = 3.24, *P* < 0.05], on number of lever-responses. Number of lever-responses during the last session of the extinction responding phase was virtually identical in the three rat groups subsequently treated with 0, 100, and 500 mg/kg *P. vulgaris* dry extract (**Figure 1**, bottom).

Under vehicle condition (0 mg/kg *P. vulgaris* dry extract), presentation of the stimulus complex robustly reinstated lever-responding: the number of lever-responses averaged indeed 90.8 ± 12.8 and was approximately five times higher than that recorded in the same rat group during the last session of the extinction responding phase (*P* < 0.0005, Newman–Keuls test; **Figure 1**, bottom). Administration of *P. vulgaris* dry extract resulted in a substantial reduction in lever-responding: although still significantly elevated in comparison to data collected in the last session of the extinction responding phase, lever-responding was indeed reduced by 40 and 45% in the rat groups treated with 100 and 500 mg/kg *P. vulgaris* dry extract, respectively, in comparison to vehicle-treated rat group (*P* < 0.005; Newman–Keuls test; **Figure 1**, bottom).

## Discussion

Presentation of different gustatory, olfactory, auditory, and visual stimuli, previously associated to the availability of a chocolate-flavored beverage, powerfully reinstated seeking behavior (i.e., unreinforced lever-responding) for the chocolate-flavored beverage in rats in which lever-responding for the chocolate-flavored beverage had been initially established and then extinguished. The high number of lever-responding recorded in the vehicle-treated rat group during the reinstatement session (i) is consistent with values observed in previous studies conducted using the same experimental procedure (Zaru et al., 2013a; Maccioni and Colombo, 2016), (ii) reflects the motivation of rats to seek for the chocolate-flavored beverage after a prolonged period of abstinence, and (iii) is conceived to model several aspects of loss of control over palatable foods and episodes of relapse into disordered eating of palatable foods in humans (see Martín-García et al., 2011; Calu et al., 2014; Maccioni and Colombo, 2016).

Acute treatment with *P. vulgaris* dry extract resulted in a remarkable reduction in number of lever-responses during the reinstatement session, suggesting that this treatment effectively attenuated the development of reinstatement of seeking behavior for the chocolate-flavored beverage. Even the lowest dose tested (100 mg/kg) was highly effective, producing a reduction in lever-responding (40% in comparison to vehicle condition) that did not differ largely from reduction produced by the highest dose tested (500 mg/kg); the comparable magnitude of the reducing effect of the two tested doses is suggestive of a saturable mechanism underlying the action of the *P. vulgaris* dry extract. These data contribute a further element to the behavioral characterization of the anorectic profile of this *P. vulgaris* dry extract: besides reducing regular food intake and body weight, intake and reinforcing properties of highly palatable nourishments (Carai et al., 2009; Fantini et al., 2009; Maccioni et al., 2010; Loi et al., 2013), this extract also reduces relapse-like behavior for a highly palatable, hedonic food.

Reduction in lever-responding during the reinstatement session was likely not due to any unspecific, sedative effect or possible malaise induced by *P. vulgaris* dry extract. A previous study (Fantini et al., 2009) demonstrated indeed that doses up to 500 mg/kg of this *P. vulgaris* dry extract failed to affect, even minimally, spontaneous locomotor activity in Wistar rats exposed to an open-field arena (a parameter highly sensitive to alterations in the state of well-being of rats).

Extracts from *P. vulgaris* are thought to exert their anorectic effects *via* two different mechanisms (both mediated by the active ingredients, lectins): (1) inhibition of the pancreatic enzyme, α-amylase, resulting in (a) suppression of starch metabolism and, in turn, decrease in glycemia and (b) delay of gastric emptying and, in turn, promotion of feelings of satiety (Jain et al., 1989, 1991; Tormo et al., 2004, 2006; for review, see Astell et al., 2013); (2) phytohemoagglutinin-stimulated release from the gut epithelial cells of the gastroenteric hormones, cholecystokinin (CCK), and glucagone-like peptide, known to contribute to the central regulation of appetite, satiety, food consumption, and hedonic eating (Carai et al., 2009; for review, see Astell et al., 2013). Since lever-responding was completely unreinforced in the reinstatement session, and no chocolate-flavored beverage was made available and ingested, the possibility that reduction in lever-responding induced by *P. vulgaris* dry extract during the reinstatement session was secondary to any inhibition of carbohydrate metabolism can reasonably be ruled out. Conversely, it is most likely that suppression of reinstatement of seeking behavior for the chocolate-flavored beverage was mediated by some central mechanism(s) regulating the rewarding and hedonic properties of food. Taking into account its role in the reinstatement of seeking behavior for cocaine (Lu et al., 2002) and morphine (Wen et al., 2013), the CCK system is a likely mediator of this action. Alternatively to lectins, a currently undisclosed ingredient of the *P. vulgaris* dry extract might be responsible for its central effects. Future studies will challenge this hypothesis.

## Conclusion

The results of the present study indicate that acute treatment with a standardized extract of beans from the functional food, *P. vulgaris*, prevented reinstatement of seeking behavior for a highly hedonic chocolate-flavored beverage in rats. These data extend to a relapse-like behavior the capability of this *P. vulgaris* dry extract to ameliorate several behaviors motivated by food (including palatable food) in laboratory rodents.

## Author Contributions

IL, GC, GG, and PMa conceived the study and designed the experiment. GC and PMa set up the experimental procedure. MC managed the literature search and summaries of previous related work. AR and PMo prepared and analyzed the plant extract. IL, VP, and PMa conducted the experiment. IL, MC, and PMa analyzed the data. GC, GG, and PMa drafted the manuscript. All authors contributed to and approved the final draft of the manuscript.

## Conflict of Interest Statement

The authors declare that the research was conducted in the absence of any commercial or financial relationships that could be construed as a potential conflict of interest.
